# Community Empowerment Under Powerful Government: A Sustainable Tourism Development Path for Cultural Heritage Sites

**DOI:** 10.3389/fpsyg.2022.752051

**Published:** 2022-02-22

**Authors:** Beiming Hu, Furong He, Lingshan Hu

**Affiliations:** ^1^School of Business Administration, Guizhou University of Finance and Economics, Guiyang, China; ^2^School of Management, China West Normal University, Nanchong, China; ^3^School of Management Science and Engineering, Nanjing University of Information Science and Technology, Nanjing, China

**Keywords:** community participation, tourism empowerment, sustainable tourism development, government control, cultural heritage sites

## Abstract

Community participation is the core of sustainable tourism development; however, it encounters obstacles at government-controlled heritage sites in China. This paper examines the status quo of community participation and residents’ empowerment perception through 25 in-depth interviews and 168 questionnaires in the Miao ethnic heritage site of Xijiang Village in southwest China, the findings reveal that: (a) The phenomenon of disempowerment focuses on the political and economic aspects, rather than the social and psychological aspects; (b) Spatial difference affects empowerment perception; and (c) Residents desire more political and education empowerment. On this basis, the present research puts forward a comprehensive empowerment system consists of system, information, and education empowerment which contributes to the theorization of community empowerment between Chinese and Western scholars and provides an alternative path to sustainable tourism development for developing countries’ cultural heritage sites.

## Introduction

Community participation is the key issue for tourism development of heritage sites and is a process that is vital to enhance long-term sustainable heritage management (Landorf, 2009). The participation of local residents in heritage sites improves residents’ sense of belonging, facilitates the development of social networks, makes a positive contribution to the quality of life of local residents and makes heritage site conservation programs more sustainable (Gursoy et al., 2002; Nicholas et al., 2009; Sirisrisak, 2009; Rasoolimanesh et al., 2017). Especially after the approval of the United Nations Educational, Scientific and Cultural Organization (UNESCO) recommendation on the world heritage convention, community participation is recognized as a fundamental tool in heritage management practices, which may enhance the quality of life and well-being of communities concerned (UNESCO, 2019). While community empowerment is the premise and guarantee of community participation. Without empowerment, community participation loses its support and significance (Tang and Wang, 2017).

Solomon (1976) marked the formal birth of the practice of empowerment in social work. Since then, “empowerment” has been introduced into sociology, communication, and pedagogy, among other fields. Western researchers consider “empowerment” to be a process of “eliminating the sense of powerlessness” (Zimmerman, 1990). Empowerment were firstly introduced as planning concepts in tourism research, and have been considered as important methods to achieve sustainable tourism development ever since. The use of empowerment in tourism research has basically followed the definition of empowerment in sociology and repeatedly argument (Joo et al., 2019). Rappaport (1985) proposes three levels of empowerment in tourism communities: personal, organizational, and community-level. Wallace and Pierce (1996) further demonstrate the implementation of information empowerment and education empowerment. After that, a four-dimension empowerment framework—including political, economic, psychological, and social empowerment was proposed (Scheyvens, 1999) and became the foundation for further studies (Nassani et al., 2019; Abou-Shouk et al., 2021).

In recent years, most scholars have begun to pay attention to the specific issues of community tourism empowerment: the empowerment of vulnerable groups of women in the community (Movono and Dahles, 2017; Nassani et al., 2019; Vujko et al., 2019; Abou-Shouk et al., 2021); the specific path of community empowerment, such as information empowerment (Cole, 2006), economic empowerment (Rayviscic and Cantoni, 2015; Boley et al., 2018) and the study of community empowerment, residents’ attitudes toward tourism development, and the effects of community tourism empowerment (Boley et al., 2014). For example, Akama (1996) clearly states that to achieve sustainable development of ecotourism in Kenya, it is necessary to empower community residents and the application of the dimensions of empowerment of local tourism stakeholders provides the grounds for the participation of local tourism stakeholders in the process of sustainable development of tourism (Naser and Saeideh, 2020). Khalid et al. (2019) implied that high community empowerment enables the community to establish successful sustainable tourism development through local people’s support for tourism and do affect the sustainability of rural tourism development by participate in decision making, empowerment, and community knowledge about tourism and has being a way to develop the sustainable tourism village in an emerging country (Fong and Lo, 2015; Purnomo et al., 2020).

However, community participation faces a variety of challenges in China, such as focus on economic interests, residents’ passive and ineffective participation, and the distribution of tourism profits and gains unfairly (Zuo, 2009; Guo, 2010; Zuo and Bao, 2012). These phenomenons exist not only in China but also in Zimbabwe and other developing countries (Yanes et al., 2019; Li et al., 2020; Gohori and Van, 2021). So Chinese scholars have identified a gap between western theories and their implementation in Chinese society though conducting research on disempowerment and the perception of empowerment in tourism communities (Zuo and Bao, 2008; Weng and Peng, 2010; Hu and Lei, 2014; Wang, 2018a). The reasons for the failure of community participation in Chinese tourism development are “the failure of rights, the lack of opportunities and ability,” and the primary cause is that the theory of community participation does not answer the question of “why to participate” from the political view; the question of “how to participate” and “being able to participate” are fully ineffective (Zuo and Bao, 2012).

Although the practice of community tourism empowerment in China has encountered many problems, it has still achieved positive impact on community participation and justice perception and promote the economic growth of heritage sites, which varies from the different management models and the state-owned private heritage management model has the highest performance (Ma, 2015; He et al., 2019). In China, heritage sites are divided into three types according to various stakeholders in China: government-controlled sites, community-autonomous sites, and franchised sites (Hu and Lei, 2014). The Table 1 described the definitions, characteristics, and representatives sites of the three management types.

**Table 1:** The table of the three management types of sites.

Management type	Features	Represent sites
Government-controlled	The government possess the ownership and use rights of the heritage tourism resources and manages the sites either directly or indirectly through its agencies	Jiuzhaigou World Heritage Site; Dujiangyan Irrigation World Heritage Site; Xijiang Miao Village
Community-autonomous	The main body of the development of heritage tourism, developing the development mode of tourism through the establishment of community autonomous institutions	Longji Rice Terraces; Langde Miao Village
Franchised	The transfer of the management right of the heritage resources, refers to the separation of the ownership and management right of the heritage resources, and the government authorizes the development and construction right, management right and income right of the heritage resources within a certain period of time to the tourism investors	Bifeng Gorge; Huangshan scenic area

The previous studies have innovated ways to achieve empowerment in tourism communities and paid special attention to system empowerment (Guo, 2010; Wang, 2012, 2018b; Chen et al., 2017). Most studies either propose a rough idea of empowerment from a micro-resident perspective or they define empowerment only from a macro-policy perspective, lacking micro-demand analysis, and measures of implementation. And few research analyzes its tourism community participation from the perspective of heritage management system, let alone focus on the powerful government place like China.

Therefore, the author believes that empowerment in tourism communities is a systematic project, which cannot be accomplished through a single method. It requires multilevel paths to achieve sustainable development of tourism empowerment. In order to effectively realize community participation, community empowerment must be carried out, especially, in western minority areas of China which are facing the reality of backward economic foundation, imperfect legal system, lack of democratic consciousness, and low quality of residents. The present research examines the status quo of community participation and explores the challenges and difficulties community empowerment has met in a government-controlled heritage site, Xijiang Miao Village, through the lens of both local and global tourism empowerment literature, as also as digs the reasons behind these obstacles deeply. With that, a reasonable and effective mechanism has been constructed to solve these problems in government-controlled heritage sites which is suit the Chinese situation.

We hope to contribute to the literature on tourism community empowerment for government-controlled heritage sites in the following ways. First, our research reaffirms the significance of western empowerment theory, while suggesting a comprehensive framework based on the features of Chinese society, which helps to enrich the theoretical system of tourism community empowerment. At the same time, We puts forward a comprehensive empowerment system consists of system, information, and education empowerment, which contributes to the theorization of community empowerment between Chinese and Western scholars and provides an alternative path to sustainable tourism development for developing countries’ cultural heritage sites. Last but not least, based on the case of Xijiang, we not only put forward a specific path for the community tourism empowerment in Xijiang Miao Village, but also provide a choice way to develop tourism in similar tourism heritage sites.

## Theoretical Framework

**Figure 1:**
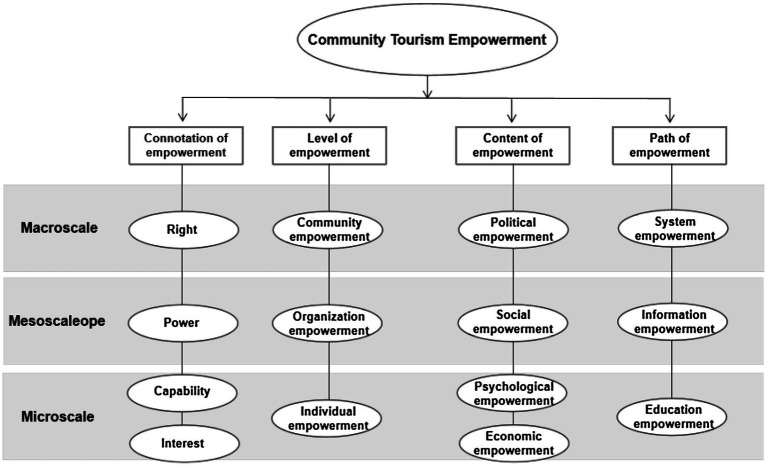
Theoretical framework of community empowerment in China.

In the West, community empowerment theory is a result of the democratic political, economic, and cultural system, which advocates human rights, private property ownership, and stakeholder theory. However, the concept was amended in China due to the public land ownership system, weak democratic awareness and participation, and the lack of Non-Governmental Organizations (NGOs; Sofield, 2003; Zuo and Bao, 2012). It is different from Western theory in three aspects. Based on the three differences of Chinese and Western community empowerment theory, this paper builds a community empowerment framework under the circumstance of Chinese society (Figure 1) to further improve the theory of community empowerment in the Chinese situation, and become the theoretical framework of this paper.

Firstly, the connotation of community empowerment differs. The western community tourism empowerment theory limits “empowerment “to “power” and “capability,” advocates the promotion of “power and capability” of community residents to participate in tourism through community empowerment, and defines this “power” as the improvement of “power” and “capability” of community residents (Sofield, 2003). Western theory provides measurements for residents to participate in community affairs (Weng and Peng, 2014), but it does little to answer the fundamental question of “Why should they participate?” The authors believe that to fully exert community participation, residents should be given rights at the macro law-making level, power at the meso-administration level, and individual capability, as well as the protection of the “interests” of community residents (the first column of Figure 1). Right is the concept of law, is the right to safeguard interests, such as human rights, property rights; while power is a political concept, which refers to the coercive power entitled to control others, such as the power of state and position.

Secondly, the object of empowerment is different. Western theory views individuals as participants in social organizations, fighting for personal right as the main method of community empowerment (Zimmerman, 1990). However, Chinese society prioritizes collective rights and interests over personal ones due to its philosophic belief in harmony between heaven and man. Therefore, individual empowerment cannot be realized if organizational and communal rights and interests are not achieved. Under this circumstance, the community becomes the representative of its residents. Only when the entire community gains legal competency can each individual participate in tourism and gain profit from it. So this theoretical framework proposes three levels of empowerment in tourism communities: personal, organizational, and community-level (column 2 of Figure 1) which is same to the Rappaport (1985), but in the Chinese context, more emphasis is placed on the systematization and top-down of the three, rather than just on a certain level.

Finally, there are various paths to realize community empowerment. Western scholars emphasize educational and informational empowerment (Wallace and Pierce, 1996), which is associated with private land ownership and democracy. Economic reform must be guaranteed by a political system which failure to ensure in Chinese community’s public interests (Boley and McGehee, 2014). In China, where social transition is still in flux, a top-down system of empowerment must be built; that is, the government needs to legitimatize community empowerment both legally and politically. Therefore, it is necessary to bring the system empowerment into the implementation path, and form a path system of the system empowerment, information empowerment, and education empowerment (column 4 of Figure 1), which is discussed in detail in the fifth part of the article.

It can be seen based on the above discussion: (1) Formulating right, power, capability, and interest into a four-in-one component system to differentiate the connotation of empowerment; (2) Incorporating empowerment framework of Scheyvens (1999) and empowerment levels of Rappaport (1985), but the emphasis is different; (3) Proposing a systematic framework where system, informational and educational empowerment are the main paths; and (4) Categorizing all the above into three scales: macro-, meso-, and micro-scale.

## Materials and Methods

### The Research Area

**Figure 2:**
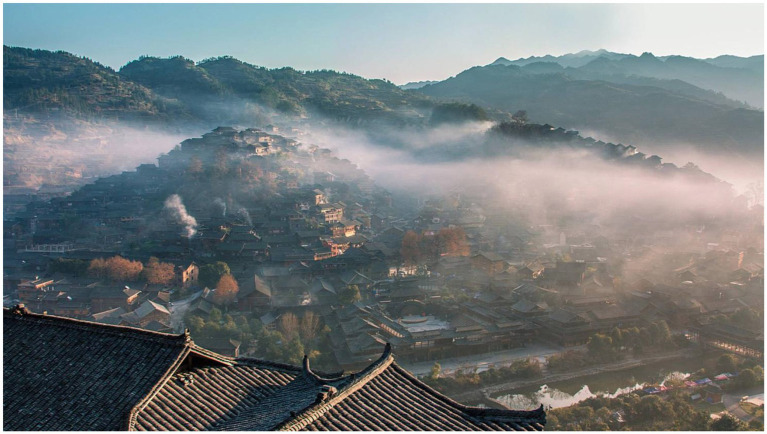
Bird’s eye-view of Xijiang Miao Village (Source: The official website of Xijiang Miao Village: http://www.xjqhmz.com/).

**Figure 3:**
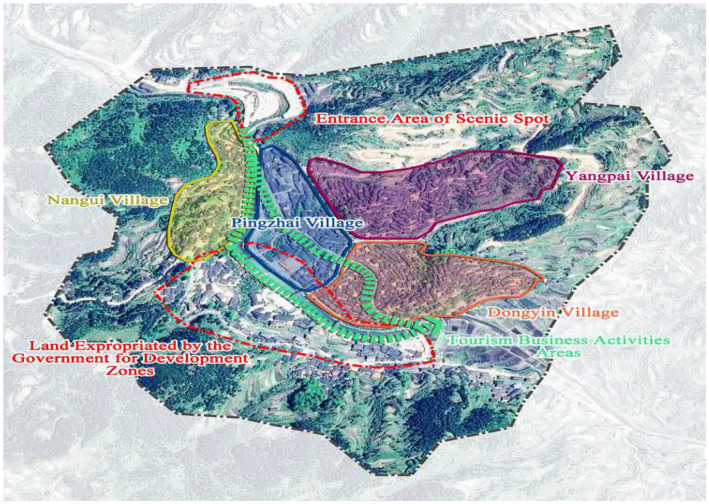
Village layout topography (drawn based on Google map).

Xijiang Miao Village, locates in the Qiangdongnan Miao and Dong Autonomous Prefecture, Guizhou Province. The Miao, an ancient cultural group and now the fourth largest ethnic minority in China, lives primarily in the mountainous areas of Guizhou, Yunnan, Sichuan, and other provinces in the southwest. Some sub-groups of the Miao, most notably the Hmong, have migrated out of China into southeast Asia and other western countries. They have their own languages, religious beliefs, and other folks. As the largest Miao inhabited area in China, Xijiang village developed during the third migration of the Miao people and now has a history of about 2,000 years and 1,288 households with a total population of nearly 6,000 accounting for 98.5% Miao globally. It is the largest Miao majority region in the world. The entire Miao habitation was built in the mountains and along water with a magnificent view of layers of terraced buildings, the typical style of Miao architecture (see Figure 2). The settlement is built in a vertical structure, with four villages: Pingzhai, Dongyin, Nangui, and Yangpai. Pingzhai and Dongyin is closer to the core business district of the scenic spot compared with the others, and the village population is larger, tourism participation is more actively (see Figure 3).

**Figure 4:**
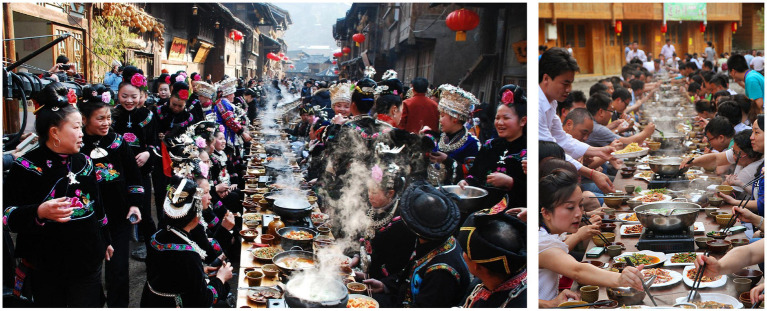
Long Table Banquet for villagers (**left**) and visitors (**right**; Source: The official website of Xijiang Miao Village: http://www.xjqhmz.com/).

Xijiang is an essential location to learn Miao history and experience Miao culture and scenery where the Miao culture is well preserved. For example, every year in the Miao calendar New Year will hold a long table banquet, a time-honored guest reception. It is reserved for special occasions, such as weddings, a baby’s first-month celebration, and important social activities among villages. At that time, each household brings out their tables and benches to be arranged in a stream on the village street, as well as provides their own hand-cooked food freely, sometimes stretching several 100 m. To the left is the host seat, while on the right is the guest seat, the host and guest sit opposite each other and sing to the wine. The host and guest are equal, and all the guests are treated equally (see Figure 4). Now, Long Table Banquet has become an important experience project for tourists in the Xijiang Miao Village.

Since its inception, it has received considerable attention from the outside world. Looking from the history of tourism development in Xijiang Miao Village, it followed a typical government power intervention path. In the first stage, the village officials took the lead to develop tourism independently, while the government took over later on and formed a new pattern which comprises of the lead of government, the intervention of enterprises and community participation. In terms of operation and management, it seems to be commercialized. However, the “Scenic Authority” and “Scenery Development Company” are both established by the government. The local residents formed the defacto ownership of tourist resources by owning their dwellings and the right to use the surrounding land. However, due to the complex interests relationships, most community residents turned from support for tourism development in the early stage to complaint about its unfairness and it is getting worse.

Xijiang Miao Village, as a typical heritage tourism destination in western ethnic areas of China, has strong representativeness in the process of interest conflict and empowerment perception. How to solve the interest conflict phenomenon of heritage tourism destination under the strong power of the government and build a harmonious and stable win-win interest mechanism are the common problems to be solved in the development of heritage tourism destination in western ethnic areas of China and other developing areas.

### Sample and Procedures

To reduce the impact of common method variance, the research group conducted two-stages surveys. In order to make the interview more meaningful and realistic and provide basic guidance for the compilation of the survey items, the research team went through expert consultation and group discussion before the investigation from July 23 to 30, 2017, and further optimized interview subjects to eventually form the in-depth interview topics of involvement, impact of tourism development, awareness, and capability which in the following Table 2. At this section, the group interviewed 25 people in total using stratified sampling, including three in the Authority, six in Pingzhai, six in Dongyin, five in Nangui, and five in Yangpai (Table 3). The number of interviews is determined by 2% of the permanent households in the four villages. Each household will be interviewed centrally. At the time of the interview, everyone of the family can participate, mainly with the opinions of the householder, and listen to the opinions of others. That is because the structure of most residents’ homes is three generations together, that is, the grandparents, the parents, and the children live together, and the children go to school, the grandparents take care of the family, and the parents are the main sources of family economy. The occupation of the interviewer on Table 3 is based on the identity of the interviewer, that is, even if some interviewers are engaged in a private shop owner, they will still run the farmland in their home, and their identity is still a farmer.

**Table 2:** In-depth interview topics.

Interview topic	Questions
Involvement	What kind of work did you participate in the tourism and how did you participate? Are you satisfied with your current job? If not, what do you want to do?
Impact of tourism development	What impact does Xijiang’s Tourism Development have on you and your family? Has income increased? Are there any changes in life? What are the changes and effects? Do you feel that you are getting a fair profit now? why?
Awareness and capability	Are you aware of major decisions in the tourism development of the scenic spot? How do you know? Are you willing to participate in the management and operation of the scenic spot? What do you think you can do?

**Table 3:** Basic information and reference number of interviewees.

No.	Reference number	Gender	Age	Education level	Family monthly income (Chinese Yuan)	Occupation
1	Scenic authority-01	F	25–44	College	6,000	Staff
2	Scenic authority-02	M	25–44	College	>10,000	Manager
3	Scenic authority-03	M	45–64	High school	6,000–10,000	Staff
4	Pingzhai-01	F	25–44	High school	3,000–6,000	Farmer (part-time staff)
5	Pingzhai-02	M	25–44	Junior high	3,000–6,000	Farmer (part-time staff)
6	Pingzhai-03	F	25–44	Junior high	3,000–6,000	Farmer (host family)
7	Pingzhai-04	M	45–64	Junior high	3,000–6,000	Farmer (part-time staff)
8	Pingzhai-05	M	45–64	Elementary	1,000–3,000	Farmer
9	Pingzhai-06	F	45–64	Elementary	1,000–3,000	Farmer
10	Dongyin-01	M	25–44	High school	6,000–10,000	Farmer (full time staff)
11	Dongyin-02	M	25–44	High school	6,000–10,000	Farmer (full time staff)
12	Dongyin-03	M	45–64	Elementary	3,000–6,000	Farmer (part-time staff)
13	Dongyin-04	F	45–64	Elementary	3,000–6,000	Farmer (part-time staff)
14	Dongyin-05	F	25–44	Junior high	3,000–6,000	Farmer (part-time staff)
15	Dongyin-06	M	45–64	Elementary	1,000–3,000	Farmer
16	Nangui-01	F	25–44	Junior high	3,000–6,000	Farmer (host family)
17	Nangui-02	M	45–64	Elementary	3,000–6,000	Farmer (host family)
18	Nangui-03	M	45–64	Elementary	3,000–6,000	Farmer (merchants)
19	Nangui-04	F	45–64	Elementary	1,000–3,000	Farmer
20	Nangui-05	M	25–44	Junior high	3,000–6,000	Farmer (merchants)
21	Yangpai-01	F	45–64	Junior high	3,000–6,000	Farmer (merchants)
22	Yangpai-02	F	25–44	High school	6,000–10,000	Farmer (host family)
23	Yangpai-03	M	45–64	Elementary	3,000–6,000	Farmer (merchants)
24	Yangpai-04	F	25–44	Junior high	3,000–6,000	Farmer (merchants)
25	Yangpai-05	F	25–44	Elementary	1,000–3,000	Farmer (part-time staff)

Since our research is funded by the National Social Science Project, the survey was welcomed and facilitated by the manager of Xijiang heritage tourism company and the “Guzangtou” who has no real power but is the informal spiritual leader of the Miao village. Our survey was led and translated by the “Guzangtou” since most interviewees cannot read or speak Mandarin. We obtained their consent orally to make sure they understood the survey was purely for research purposes, would not be released to any third-party, and conducted anonymously to protect their rights and privacy.

Based on the in-depth interviews and predecessors’ research (Chen et al., 2011; He, 2017), a questionnaire following the four-dimensional framework of Scheyvens (1999) with 15 measurement questions were retained using a five-point Likert scale after several rounds of intense group discussions (Table 4). From February 10 to 17, 2018, the trained research team conducted on the spot surveys with 220 villagers in four villages with the 2% of the number of permanent residents, and 168 valid questionnaires were obtained which is conducted when the villagers’ meetings were held in each village.

**Table 4:** Measurement scale of community empowerment.

Dimensions	Items
Political empowerment	Participation channels: Residents have channels to represent their opinions.
	Political rights: My opinions are valued by the Scenic Authority.
	Participation opportunities: I can participate in the planning, decision-making, and management of tourism areas.
	Community organization participation: The village committee can represent the rights and interests of residents.
Economic empowerment	Income increase: The development of tourism has brought me a stable income.
	Income sources: My main source of income is from tourism development.
	Job opportunities: Tourism development provides us more employment opportunities.
	Life improvement: The development of tourism has clearly improved our standard of living.
Social empowerment	Neighborhood relations: Tourism development makes relations with neighbors more intimate.
	Community cohesion: The development of tourism enhances our collective identity.
	Women’s status: Our status as women has improved.
	Public services: The level of community public services has greatly increased.
Psychological empowerment	Pride: I feel proud as a member of Miao Village.
	Self-confidence: I have more confidence in future life.
	Education accessibility: There are more opportunities to receive various education and training.

We used software SPSS 20.0 for mean and SD of the measurement items and Cronbach’s alpha indicator to test the reliability and validity of the survey. The overall Cronbach’s α coefficient was 0.8868, and the Cronbach’s α coefficients of economic, psychology, social, and political dimensions 0.8178, 0.8973, 0.9125, and 0.9198, respectively. Reliability is generally acceptable if Cronbach’s α coefficient value is greater than 0.7, high if greater than 0.9. The results of our survey are greater than 0.7 and close to 0.9 standard, indicating a high reliability.

## Results

### Political Disempowerment and Empowerment Perception

With the guidance of local village leaders and cadres, residents participated in tourism independently and autonomously. Constrained by underdeveloped infrastructure, local tourism evolved fairly slowly. In 2007, local government became fully involved in its tourism development and funded planning, infrastructure construction, marketing, and personnel training. It is undeniable that local tourism developed rapidly after governmental involvement. By 2017, the number of tourists had reached 7.5 million. There are currently more than 100 Miao restaurants, 500 hotels and inns, and 160 souvenir shops in Xijiang.

Significantly, the government is the main investor, marketer, and manager at the same time in Xijiang. The tourism development company and the heritage site administration committee take charge of Xijiang’s tourism, which are set up by the government with the same staff. Therefore, the government takes care of all the issues of the development of the scenic spot and determines the development direction of the scenic spot. But the actual owners of the cultural and scenic capital of Xijiang are residents, so their ownership position has been taken away by the government.

Their development goals and rights are not fully satisfied in three main ways. First, residents lost ownership of their land due to the mandatory land transfer system. In order to expand the commercial space within the heritage site, the management company, in the name of government, forced the requisition of several acres of fertile land along the Baishui River (the main river channel in Xijiang), and they converted it into commercial buildings for rental and tax purposes (Figure 3). High profits were made through this process, but residents received only a modest one-time land compensation.

Second, residents have little access to information and few rights to express themselves. During the development, all information related to planning, construction, financing, and personnel are completely closed to the community. Even income distribution is formulated by the government without notice. Finally, residents’ right to education is not well-protected either. Training and education are important guarantees for the development rights of community residents; however, survey statistics show that 60% of interviewees only have primary and secondary education and that 80% of villagers do not directly participate in tourism management or service work. As the residents say:

*“Can we have any opinions on the scenic spot? Even if we do, who can we report to and who will listen to us?”—(Pingzhai-05).*

*“Let the local people participate in the management of our scenic area? Why should they be involved? Do they understand heritage protection? Do they understand how to promote the scenic area?”—(Admin-02).*

These statements coincide with arguments of Scheyvens (1999) which pointed out that political disempowerment exists, where the management agency is a tyrannical, self-interest-oriented leadership organization. They believe it is because of their active charity that community residents benefits from tourism development. Community members feel that they have little or no opportunity and power to express relevant opinions, and that they are not allowed to participate in the decisions of tourism development.

The research on the perception of political empowerment further confirms this problem, which results show that community organization participation contributes the most to political empowerment perception, while limited opportunity to participate in decision-making is the biggest obstacle for empowerment in all four villages (Table 5). In general, Nangui and Yangpai have higher political perception than the other two villages that are closer to the main tourist area. It seems surprising that the more active residents are in tourism-based economic practices, the lower their perception of political empowerment is.

**Table 5:** Means analysis of political empowerment perception.

Item	Pingzhai	Dongyin	Nangui	Yangpai	Total
Participation channels	1.796	1.829	2.268	2.122	2.007
Political rights	1.779	1.786	1.968	2.012	1.896
Participation opportunities	1.545	1.468	1.323	1.345	1.416
Community organization participation	2.029	2.121	2.346	2.453	2.227

Based on a comprehensive analysis of interview and survey results, the study found that the more residents are dependent on the tourism economy and exposed to the outside world, the more they know about their rights and thus have more conflicts with the government. On the contrary, the less participation and access to information, the less the conflicts with the government. Ironically, this situation makes them believe that they have higher political empowerment.

### Economic Disempowerment and Empowerment Perception

Xijiang residents profited more following the increase in tourism. There are mainly four ways to make money: (1) Independent investment and operation: residents use their own property to run ethnic restaurants, inns, stores, etc.; (2) Rental: most residents rent their property to tourists for accommodation due to a lack of funds and skills to produce other revenue; (3) Selling local products and ethnic handicrafts such as Miao medicine, embroidery, silverware, etc.; and (4) Participating in labor-intensive services, such as song and dance performances, cleaning, car battery transportation, etc.

However, conflicts appear gradually accompanied by the distribution of interests. As the government is not an “economic entity” in a complete sense, it needs financial support to achieve some of its political goals. Hence, it is inevitable that the government fights with community residents for economic resources.

For example, the government claimed that it stipulated the cost of infrastructure construction and marketing expenses for the development of scenic spot, so most of the ticket income (90%) is obtained by the scenic spot management company on behalf of the government, while only 10% of the profit went to the residents in the form of a completed heritage preservation project. What is more serious is that residents have very little agency in the way of income distribution, which will certainly arouse the dissatisfaction of the community residents. Just like the residents say:

*“We do have better income and better living standard than before. We receive thousands of RMB Yuans each year from the government, but most of the money is earned by the outside business owners. My family only collects some rent every year.”—(Dong Yin-04).*

*“Yes, we can make more money than just working in the farm in the past. But our family is on the mountain, the business is only good on holidays and during July and August. We can make more money during these times but the business is much worse at other times. The outside business owners are not willing to rent our house.”—(Yangpai-03).*

Local residents gain continuous economic benefits from tourism industry and their living standards are significantly improved. But they have obtained only a small amount of unstable income while community elites, foreign tourism developers, and local governments have obtained most of the benefits. This phenomenon is the economic disempowerment described by Scheyvens (1999).

Even with the internal and external conflicts of interests, most respondents agree that tourism development in Xijiang has brought real benefits to the local community, such as increased income, more job opportunities, and better standards of living (Table 6).

**Table 6:** Means analysis of economic empowerment perception.

Item	Pingzhai	Dongyin	Nangui	Yangpai	Total
Income increase	4.767	4.865	4.021	4.126	4.447
Income sources	4.876	4.789	4.131	4.154	4.516
Job opportunities	4.445	4.342	4.023	4.112	4.243

A phenomenon worthy of attention is that compared to Pingzhai and Dongyin Villages which are closer to the core tourism area, Nangui Village and Yangpai Village have relatively lower perceptions of economic empowerment. But Pingzhai and Dongyin benefit more due to their advantageous location. In this case, spatial differences influence residents’ perception of economic empowerment, but behind the scenes, the imbalanced income distribution system is key.

### Social Disempowerment and Empowerment Perception

The social empowerment in tourism is realized when tourism maintains or improves the balance of the local community; when individuals and families work together to build a successful tourism company and the degree of integration of the community is improved; when partial tourism proceeds are used on community development (Scheyvens, 1999). Tourism development has greatly enhanced the collective sense of honor and community cohesion of Miao people in Xijiang, where the perception of community cohesion, the status of women, and the level of public services are all positive in the four villages (Table 7). They spontaneously established the “Family Inn Association” and the “Xijiang Senior Citizens Association.” The development of the tourism industry combines traditional culture and interpersonal relationships in the ethnic areas and strengthens the cultural memory of the community (Chen et al., 2013).

**Table 7:** Means analysis of social empowerment perception.

Item	Pingzhai	Dongyin	Nangui	Yangpai	Total
Neighborhood relations	4.624	4.679	3.012	3.343	3.913
Community cohesion	4.523	4.443	4.044	3. 908	4.228
Women’s status	4.463	4.767	4.789	4.768	4.708
Public services	4.798	4.754	4.786	4.864	4.798

Nevertheless, most touristic activities happen along the main street of the most scenic area in the region. Thus, the two villages in the core area, Pingzhai and Dongyin, have increased chances to receive tourists, while the other two villages, Nangui and Yangpai, have less opportunities (see Figure 3). For example, property rental and business are more prosperous in Pingzhai and Dongyin than in Nangui and Yangpai, while the negative economic impacts of tourism, such as rising living costs and shortages of goods and services, are equal among all villages. Increasing inequality between the poor and the rich generates tension among community residents. In the off-season, they even fight for opportunities to receive tourists; thus, relationships in the community have deteriorated. But before the tourism development, the spatial differences of villages’ location will not bring obvious economic level differences. Neighbors had closer relationships than now and many kinds of mutual help phenomenon exist, the community cohesion was strong. Just they say:

*“We are old and we are female. We sweep the streets for living. Most of the time we sell things here. The money is definitely less, but we can only do this!”—(Yangpai-05).*

*“We have more income and better living standard than before, but the relationship between relatives and neighbors is not as good as it used to be. We used to practice Long Table Banquets in the stockades before. It was not for outsiders. It is for our a good relationship. Now everyone is money-driven and often quarreled.”—(Nangui-04).*

### Psychological Disempowerment and Empowerment Perception

As indicated in Table 8, residents have a stronger perception of “pride” and “self-confidence,” which are beyond their words in the interview. As mentioned, Xijiang Miao Village has been acknowledged nationwide and residents’ sense of ethnic pride has greatly improved.

**Table 8:** Means analysis of psychological perception.

Item	Pingzhai	Dongyin	Nangui	Yangpai	Total
Pride	4.445	4.543	4.243	4.342	4.396
Self-confidence	4.478	4.564	4.043	4.126	4.305
Education accessibility	3.698	3.543	3.786	3.584	3.650

*“Who would have known in the past that we will engage in tourism in this place. Some of them (tourists) like our shabby houses. We must thank the government for developing tourism!”—(Pingzhai-03).*

*“There are more time to dance and sing, and host festivals. People outside like to watch us dance and hear us sing. We also like it.”—(Dongyin-05).*

Every informant confirmed that with the increase of family income and the improvement of living standards, residents’ self-confidence in the future was strengthened. According to Scheyvens (1999), psychological empowerment is established when foreign tourists consider the local culture, natural resources, or traditional knowledge to be valuable and unique, which in turn increases the pride and self-esteem of local residents because they feeling that community is concerned and there are special things to share with outsiders. The increasing confidence encourages them to receive further education and training. Based on this opinion, Table 8 gives us a warning: compared to their perception of “pride” and “self-confidence,” their perception of “opportunity for education” is weaker, which reflects the urgency of increasing education empowerment in ethnic minority areas.

They actively seek various education and training programs to increase employment opportunities. In the process of tourism development, local women gained more income from tourism development than traditional agriculture and demonstrated their capabilities by participating in singing or dancing performances. Thus, their family status has also been improved.

## Conclusion and Discussion

### Research Conclusion

From the above analysis, Xijiang Miao Village, as a typical government-controlled ethnic heritage site in western China, manifests the features of compulsory government-led tourism development. The results for field research demonstrated that: (1) The disempowerment phenomenon concentrate on the political and economic aspects not on social and psychological aspects. Most interviewees showed great respect of local culture and satisfaction of their job arrangements in the administration bureau, and they has shared the economic benefits and improved living standards through tourism development, but complaints about imbalanced interests distribution still exist and the lack of political participation has become the biggest obstacle to the empowerment of residents’ communities. (2) Spatial difference affects the degree of participation and further influence empowerment perception. Pingzhai and Dongyin Villages, which are closer to the main street and have more opportunities to participate in the tourism development, have a lower perception of political empowerment, while have higher economical, social, and psychological empowerment perception, but it does not work in the villages of Nangui and Yangpai. (3) Residents desire more political and education empowerment. Residents’ weaker perception of political empowerment reflects the potential growth and necessity of political empowerment in government-controlled heritage sites like Xijiang Miao Village. This shows a stronger desire for political empowerment in villages that are more economically active, while education empowerment is the fundamental way to achieve it.

Such research conclusions have been drawn in other similar settings, Abou-Shouk et al. (2021) with partial least squares structural equation modelling (PLS-SEM) employed to analyze the perceptions of 784 respondents across the three countries and the findings reveal that significantly different effects of women’s empowerment on tourism development and must not ignore socio-cultural facets of empowerment (Aghazamani et al., 2020), Thanks to being given information and knowledge through empowerment, the villagers set up confidence and responsibilities to participate, which has changed their cognition of, emotion for and interaction with community planning, development and themselves (Xu et al., 2019). At the same time, Ostrom and colleagues consider non-binding communication can serve as an effective mechanism for solving social dilemma problems in common-pool resource settings (Ostrom, 2008; Ahn et al., 2010).

### Discussion

Studies and practices have proved the importance of taking residents’ will and interest into consideration during tourism development in ethnic areas (Wang, 2006). The current system has disempowered community residents to a certain extent, thus as described in the theoretical framework in Figure 1, the evolution from a compulsory top-down system to an inductive bottom-up one, which emphasizes the leading position of local residents and unanimous recognition and participation, is necessary. In all, community residents need a comprehensive empowerment system to secure their rights and interests.

Xijiang Miao Village demonstrates the common phenomenon that many ethnic heritage sites in western China face. With the development of tourism, the economic, social, and psychological empowerment of community residents has been realized partly, but this kind of government-led top-down mandatory supply system of tourism empowerment has not fundamentally solved the contradictions in the process of community tourism development in ethnic areas. From the case of tourism empowerment in the Xijiang Miao village, we have found a path to from the contents to achieve “empowerment” in the community, that is, to achieve political empowerment from institutional empowerment and to achieve autonomous empowerment through education empowerment and information empowerment.

#### System Empowerment Is the Fundamental Guarantee of Community Benefits

In recent years, Chinese scholars have also explored the reasons for the failure of community participation in China from the perspective of laws and regulations, and they have proposed a basic path for system empowerment. It is apparent that current laws and regulations fail to ensure the right to community participation and cannot define, regulated or protect it (Wang, 2012; Wang and Zheng, 2015). However, real community empowerment requires the long-term support and authorization of the government which is the Chinese scholars hope to set up (Peng, 2010; Weng and Peng, 2014; Wang and Zheng, 2015). Based on the field survey and forefather’s research, the authors suggest developing system empowerment from the following three aspects.

First of all, land ownership reform is the foundation of system empowerment. In recent years, the survival and livelihood of farmers who have lost land due to tourism development have been a matter of common concern for the Chinese government. Some experts and scholars advocate returning land ownership to farmers; that is, privatizing the land (Wu, 2009). However, most scholars believe that Chinese farmers need stable land and contractual land management rights rather than land ownership (Chen et al., 2013). In fact, due to the strong power of local government and collectivism, land privatization may bring about huge institutional costs, out-of-control land sales, and other issues related to rural stability (The office of the CPC Central Committee and the Office of the State Council, 2014). In 2014, the publication of No. 71 (Document of the Bureau of National Development) formally separated land ownership, contracting rights, and management rights in rural China which identified the direction for the reform of land ownership in heritage sites. Land management rights refers to the fact that residents have legal operation and management rights to land sale, lease, mortgage, transfer and other activities, and these rights can be legally guaranteed (Zhao, 2008). In order to return the right of permanent management of land to farmers, first of all, we must ensure that the right of management become a real “property right.” Secondly, we must establish a unite, open, competitive, and orderly market for land management rights to guarantee the transfer is in order. Only when the land management right becomes a real property right, can community residents have the right to dispose their own land and management rights, as well as protect the resources related to the land. This is way, we can build institutional protection barriers for community residents in the process of tourism development.

What is more, property laws governing tourist attraction should be promulgated. Research has found that there were two basic interest conflicts between community residents, governments, and outside developers: under-compensated land price and deliberately neglected compensation of future gain of assets during the land transfer process (Sofield, 2003). In Xijiang Miao Village, the residential building, living environment (such as lakes, mountains and forests, and animal and plant resources, historical sites, etc.) and folk customs of the local community have become the core tourist attractions, although local governments and their development companies have invested in the development of tourism (such as parking lots, plazas, roads, etc.), it is not the result of these inputs that really attract tourists, but the original Miao culture and landscape. The landscape, culture, and customs are all in line with the constructivist authentic culture and retain the traditional living customs and cultural art of the Miao nationality. Yet, local residents, the actual owners of these tourist attractions, were left out from financial gain. Therefore, it is urgent to stipulate provisions on intangible rights in property law. Only in this way can we protect community residents’ rights and provide them a leading role to participate in tourism development.

Last but not least, a mutual benefit mechanism between local residents and developers should be established in any land management system. In the existing way of tourism development, land is often purchased or rent by outside investors at one time to obtain more long-term profits. It is neither fair nor sustainable for developers to benefit the most while residents receive only limited rent from land-leasing. The biggest dissatisfaction among the community residents where we surveyed is the profit imbalance. It is not poverty but inequality that caused sharp conflicts in community tourism development.

Thus, instead of renting or transferring their land, community residents should invest their land in the tourism industry for future gains which the innovative forms of land transfer are also the guidance of government. The use of land shares is a new and effective, yet rarely adopted, method to resolve conflicts of interest in land transfer which allows residents to lease their land to enterprises and also to invest their property—with tourist attractions attached to that land—into the share capital of the enterprises.

In that case, community residents can obtain stable income as well as dividends from the development of the heritage site according to the number of shares converted from tourist attractions. Besides, as a shareholder of the tourism enterprise, community residents can also exercise real rights to participate in tourism development, such as planning, decision-making, and management of tourism areas.

#### Information Empowerment Makes Community Participation Mechanism More Transparent

Information is the key to navigating decision-making among various stakeholders. Information asymmetry is the leading cause of the tragedy of the commons. Under the influence of a planned economy, with its policies and bureaucracy, local governments avoid informing local residents of their decisions in case any unpredictable incidents will jeopardize upcoming investments. Although China has achieved rapid development during economic transition, democratization, and civil rights awareness have not yet reached the same level.

The lack of a communication platform and mechanism puts residents in a vulnerable position. In order to change that, the government should first recognize residents as the main body of tourism development and increase their self-awareness. It is the government’s responsibility to initiate effective communication and truly benefit local people instead of taking advantage of them. Next, the government needs to set up a long-term mechanism for information disclosure on tourism development which specifies the time, periodicity, and content of information to be released. Finally, a platform for information exchange and communication should be built; for example, the village committee or tourism professional associations could act as liaisons so that information related to tourism development can be delivered to each community inhabitant.

#### Education Empowerment Fosters the Ability of Community Participation

In the survey data from Xijiang Miao Village, family income and community tourism participation are positively related to education level. Households that have received junior high school education and above have a higher family income and more members participating in community tourism than those who only have junior high degrees or below. It is helpful for residents to obtain more information, but this requires residents do have awareness of their rights and the ability to fight for them. Thus, education empowerment is extremely important to realize that.

Education empowerment includes three components. At first, community residents must improve their legal awareness to understand national policies on land expropriation, resources protection, and traditional culture protection. Then, modern tourists have an ever-increasing higher demand of quality from tourism professionals, which stimulate the community residents to improve their operating abilities, management abilities, and service skills. In Western developed countries, third-party civic organizations take charge of the educational and training functions for most community residents. However, in China, these organizations develop relatively slowly and lack the necessary financial support due to constraints of the social system. Therefore, it is the governments’ responsibility and obligation to provide education and professional skill training. Local governments should allocate a proportion from tourist gains to provide education and training or establish a special training fund for this purpose.

In the end, abundant cultural practices in heritage sites in western China have become important tourist attractions, such as folk songs and dances, arts, and crafts. Local governments should also allocate special funds to educate and train residents to maintain craftsmanship in the intangible cultural heritage of these peoples, such as certifying inheritors of non-material cultural heritage and providing training on cultural heritage inheritance education, especially to the younger generation so that they find confidence and recognition from their own culture. Additionally, local governments in ethnic regions should strengthen cooperation with tourism colleges and research institutions. In that case, colleges and research institutions find research places, and the voluntary lectures of experts and students’ internships in the community can greatly improve the residents’ awareness of their rights and ability to participate in local tourism development.

## Research Gaps and Outlook

This work measures the residents’ empowerment of power in social, political, and other aspects from the perspective of psychological perception. However, the means and methods of measurement have certain limitations, which cannot relieve the influence of cultural traditions and individual differences, and the source of analysis and conclusions of the Chinese political framework will inevitably lack rigor.

In the course of the study, we also found that the degree of tourism participation is different from the occupations of residents, and then the perception of residents’ empowerment varies. In the future, we can further study the differences in the perception of empowerment and government satisfaction in different professions.

Obviously, it is not possible for community residents alone to realize true empowerment in government-controlled heritage sites. So combine the research results and previous studies, this paper forms a comprehensive empowerment system consists of system, information, and education empowerment. The core components of system empowerment are reform of collective land ownership, the land leasing system, and property rights law. Additionally, we need to establish a mechanism and platform for residents to express themselves, discuss tourism related affairs, exchange information, and access policy announcements. Education empowerment is equally important which improve their capability to become fully engaged in tourism.

This research reaffirms the significance of western empowerment theory, while suggesting alterations should be made based on the features of Chinese society. The implication from the Xijiang case is thus meaningful and can be replicated at many other government-controlled heritage sites.

## Data Availability Statement

The original contributions presented in the study are included in the article/supplementary material, further inquiries can be directed to the corresponding author.

## Ethics Statement

The studies involving human participants were reviewed and approved by the Academic Committee of Guizhou University of Finance and Economics. Written informed consent to participate in this study was provided by the participants’ legal guardian/next of kin. Written informed consent was obtained from the individual(s) for the publication of any identifiable images or data included in this article.

## Author Contributions

BH conceived the research and wrote the original draft. FH participated in the revision of the manuscript. LH collated the data. All authors contributed to the article and approved the submitted version.

## Funding

This research was funded by China National Social Science Fund Project (17BMZ130) and Graduate Project of Guizhou University of Finance and Economics (2020ZXSY24).

## Conflict of Interest

The authors declare that the research was conducted in the absence of any commercial or financial relationships that could be construed as a potential conflict of interest.

## Publisher’s Note

All claims expressed in this article are solely those of the authors and do not necessarily represent those of their affiliated organizations, or those of the publisher, the editors and the reviewers. Any product that may be evaluated in this article, or claim that may be made by its manufacturer, is not guaranteed or endorsed by the publisher.
